# Effects of dysprosium oxide addition on the structural, mechanical, optical, and radiation shielding properties of borate tellurite germanate glasses

**DOI:** 10.1038/s41598-025-16662-6

**Published:** 2025-11-29

**Authors:** Kawa M. Kaky, M. I. Sayyed, K. A. Mahmoud, M. H. A. Mhareb, Abed Jawad Kadhim, Sudha D. Kamath

**Affiliations:** 1 Al-Nisour University College, Baghdad, 10012 Iraq; 2 Department of Physics, Faculty of Science, Isra University, Amman, 11622 Jordan; 3 Renewable Energy and Environmental Technology Center, University of Tabuk, 47913 Tabuk, Saudi Arabia; 4 Department of Physics and Technical Sciences, Western Caspian University, Baku, Azerbaijan; 5 R&D Office, Vice Presidency for Scientific Research and Innovation, Imam Abdulrahman Bin Faisal University, P.O. Box 1982, 31441 Dammam, Saudi Arabia; 6 Ural Federal University, 19 Mira St, Yekaterinburg, Russia 620002; 7 Department of Physics, College of Science, Imam Abdulrahman Bin Faisal University, P.O. Box 1982, 31441 Dammam, Saudi Arabia; 8 Basic and Applied Scientific Research Center, Imam Abdulrahman Bin Faisal University, P.O. Box 1982, 31441 Dammam, Saudi Arabia; 9 Department of Physics, Manipal Institute of Technology, Manipal Academy of Higher Education, Manipal, Karnataka India

**Keywords:** Dysprosium oxide, Borate-tellurite-germinate glass, Optical properties, Radiation shielding properties, Gamma ray, Materials science, Physics

## Abstract

The current work seeks to fabricate a series of four bulk glasses using the melt-quenching method according to the chemical formula of (35-x) B_2_O_3_ + 10GeO_2_ + 20TeO_2_ + 35MgO + x Dy_2_O_3_, where x = 1.25, 2.5, 3.75, and 5 mol%. XRD detected the amorphous nature of the fabricated glasses with a 2θ range from 10 to 80°, whereas the glasses’ absorption spectra were explored in the 350–1000 nm wavelength region. The band gap (E_g_) values were calculated using Mott and Davis’s concept while calculating several optical parameters, including the optical basicity (Λ), reflection loss (R), optical electronegativity (χ), electron polarizability (αₒ), metallization, and transmission (T). The impact of substitution of B_2_O_3_ by Dy_2_O_3_ on the mechanical properties was examined utilizing the Makishima and Mackenzie method. Moreover, the prepared glasses’ γ-ray shielding was found via a Monte Carlo simulation. The simulated data showed that the increase in B_2_O_3_’s partial substitution by Dy_2_O_3_ content enhances the prepared glasses’ shielding properties. The highest linear attenuation coefficient in the current study was achieved for the 5 mol% Dy_2_O_3_-doped glass sample, where its LACs decreased over the range of 152.849–0.124 cm^−1^, with the gamma-ray energy raised throughout 0.015–15 MeV, respectively.

## Introduction

One of the tools used often in the hospital for patients treatment is gamma radiation, particularly in the radiology, radiography, and radiotherapy units. Gamma radiation is vital in the treatment of cancers and the diagnosis of sicknesses via imaging techniques, confirming that it cannot be marginalized in our day-to-day activities. Gamma radiation is the highest energetic type of radiation, electromagnetic wave like, massless, and highly penetrative on interactions with matter. Because of the aforementioned characteristics possessed by gamma radiation, frequent interactions with matter could result in several health challenges, among which are ARS (acute radiation syndrome), like vomiting and nausea, fatigue, diarrhea, and bone marrow damages. On excess subjection, gamma radiation can similarly result in cancer related sicknesses like lung cancer, thyroid cancer, leukemia, and breast cancer. Not only that, when humans are not controlled from radiation, they might suffer from suppression of immune system, cataracts, radiation burn, and genetic mutations. Long time exposure to gamma radiation can pose radiation-induced sicknesses such as cardiovascular desease, neurological damages, and fibrosis^1–3^.

Despite the above-mentioned advantages of gamma radiation, which make it unmarginalized, the aforementioned health challenges have made it a terrible tool that must be controlled in order to prevent people from too much exposure to it. This prevention could be achieved by means of shielding technology, which offered a radiation source–human barrier^4–6^. It is worthy of note that radiation shielding offers protection against overexposure and sicknesses induced by radiation, and most of all, it preserves biodiversity and safeguards ecosystems through prevention of radiation from air, water, and soil contamination. Radiation shielding also minimizes the costly efforts required for environmental remediation via radiation contamination prevention, and it improves the health and well-being of the population by contributing to a safer environment. Reduction in sabotage or theft of radioactive materials as well as prevention of nuclear accidents are ensured through strict shielding modalities^7–10^.

Based on the initially mentioned advantages offered by radiation shielding technology, the search for materials with better shielding characteristics is highly paramount. As a result, researchers delve into a thorough search for better materials that could serve the purpose. Many authors recommended materials like alloys, ceramics and others for shielding applications^11–14^. Concrete with an average density of 2.4 g/cm^3^ and bricks with a density ranging between 1.8 and 2.2 g/cm^3^ both offer good protection against gamma radiation with cost-effectiveness and environmentally friendly nature. Their high structural integrity makes them very strong mechanically and durable, and their flexible nature makes it possible to remold concrete and bricks into suitable shapes. The high thermal and chemical stability of ceramics makes it an effective material in terms of resistance to degradation and radiation damage.

Lead’s high density render the material a good radiation shielding candidate. Despite the advantages offered by such materials in shielding gamma radiation, these materials should be noted to possess some fallbacks, like the low attenuation of concretes at higher energy, the cracking and brittle nature of ceramics, the low-density nature of bricks compared to other materials, the problem of environmental issues and toxicity presented by lead, and the creation of secondary radiation by metal alloys as they have the ability to undergo activation. These restrictions forced the necessity for expanded research for alternative materials that can complement the aforementioned materials in shielding applications^15–17^.

To improve on the limitations of the above-mentioned materials, previous research reported that glass materials are the excellent materials that could solve such limitations and offer a promising radiation shielding performance. The lightweight, high visibility, and high transparency, among other natures of glass materials, make it a versatile and promising candidate for applications involving radiation shielding^9,18–21^. These three properties are highly vital to be considered in a glass material that must be applied in radiation protection, as such must not be compromised, and are necessarily assessed while searching for a better radiation-shielding glass material^22,23^.

Theoretically, Monte Carlo N-Particle (MCNP-5) simulation is an essential method for assessing a material’s gamma radiation shielding parameters because it gives reliable data for complex interactions^24,25^.

The present study’s objective is to develop a novel series of glass based on borate-tellurite-germanate glass formers doped with various Dy_2_O_3_ concentrations. The produced glasses’ mechanical, optical, and γ-ray shielding parameters were thoroughly investigated in relation to the partial substitution of B_2_O_3_ by Dy_2_O_3_.

## Methodology

### Sample fabrication

**Fig. 1:**
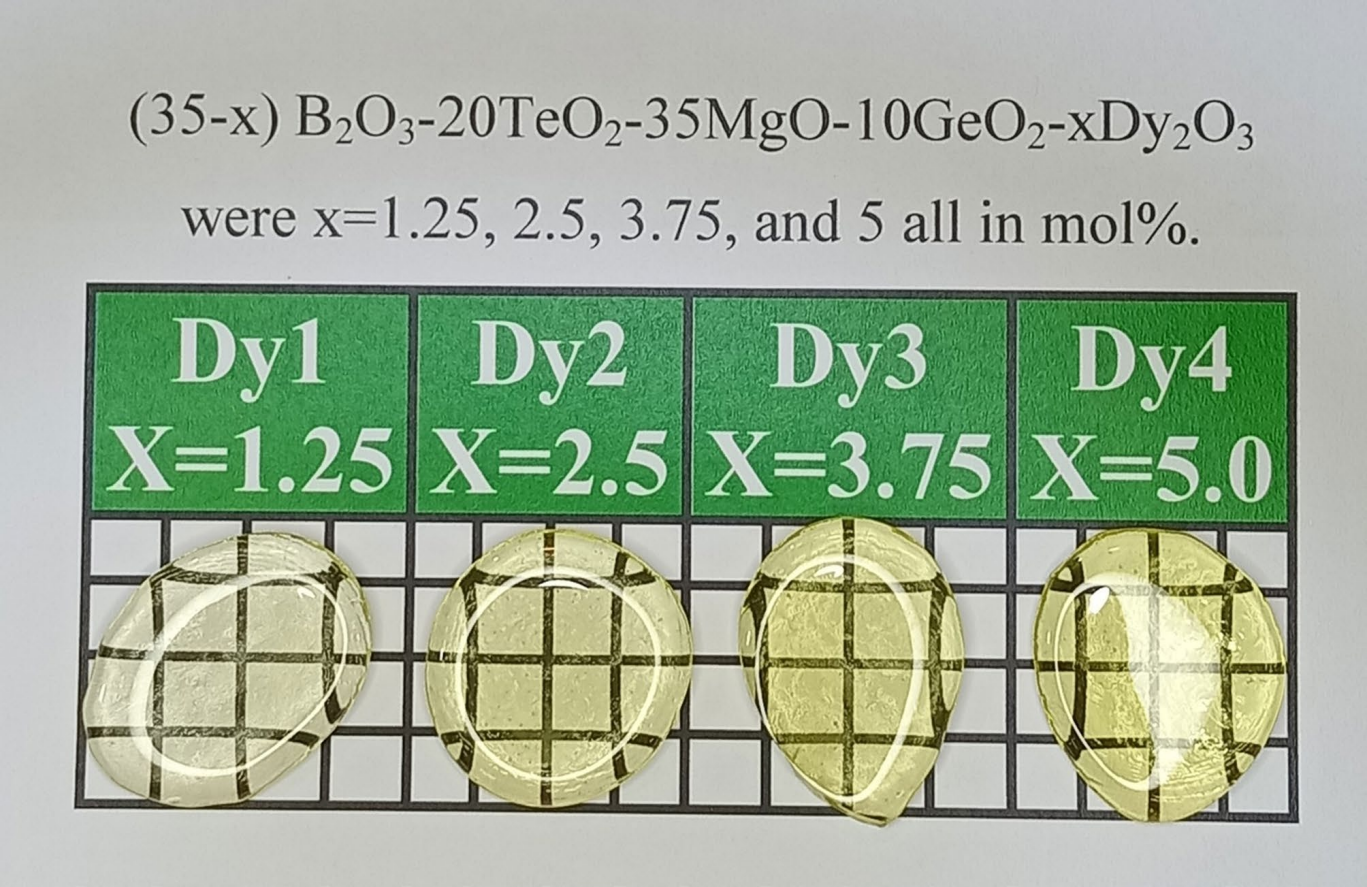
The color gradient of the fabricated Dy glasses as the Dy_2_O_3_ concentration raised within the prepared glass.

A group of four bulk glasses with a composition of (35-x) B_2_O_3_ + 10GeO_2_ + 20TeO_2_ + 35MgO + x Dy_2_O_3_, where x = 1.25, 2.5, 3.75, and 5 mol%, were fabricated by using the melting and annealing process. From Table 1, we reported the ratio for each chemical used in each sample with labels of Dy1, Dy2, Dy3, and Dy4 based on the concentration of Dy_2_O_3_. Four batches of 20 g were melted in an electric furnace for 20 min at a temperature of 1100 °C; the melt was then cast onto a stainless-steel plate and allowed to cool down gradually to avoid any sudden high change in temperature, which may lead to cracks. The samples were then moved to another oven at 350 °C and were kept there for five hours for annealing. Then, the oven was turned off to let the samples cool down to room temperature. As illustrated in Fig. 1, the addition of Dy_2_O_3_ to the G-T-B glass slightly changes the color of the prepared glasses from nearly transparent to light yellow. The substitution of B_2_O_3_ with the light yellow Dy_2_O_3_ is the main reason for the change in the color of fabricated G-T-B glasses.

**Table 1:** Glass composition for the fabricated samples.

Glass code	Chemical composition (mol%)					Density (g/cm^3^)
	B_2_O_3_	TeO_2_	GeO_2_	MgO	Dy_2_O_3_	
Dy1	33.75	20	10	35	1.25	3.7397
Dy2	32.5	20	10	35	2.5	3.8065
Dy3	31.25	20	10	35	3.75	3.8732
Dy4	30	20	10	35	5	3.9400

### Characterization

The Shimadzu XRD-6000 was used to detect the amorphous nature of the glass samples with a 2θ range from 10 to 80 degrees. Whereas the absorption spectra of glasses in the 350–1000 nm wavelength region were determined using a spectrometer, UV-vis-NIR-Shimadzu 3101. The band gap (E_g_) values were computed using Mott and Davis’s concept^26^ And the absorption edge:

1 $$\:hv\alpha\:=A(hv-{E}_{g}{)}^{n}$$

Where the absorption coefficient, constant, and photon energy are referred to by α, A, and hv, respectively, the following formula was used to calculate the refractive index (n) based on E_g_ values:

2 $$\:\frac{({n}^{2}-1)}{({n}^{2}+1)}=1-\sqrt{\frac{{E}_{g}}{20}}$$

Several optical parameters were calculated by employing various relations mentioned previously^27^; these parameters include optical basicity (Λ), reflection loss (R), optical electronegativity (χ), electron polarizability (αₒ), metallization, and transmission (T).

### Mechanical, physical, and structural properties

The density of the samples produced was ascertained employing the Archimedes concept and the below relation, as illustrated in our earlier works^28,29^:

3 $$\:\rho\:=\frac{{W}_{A}}{{W}_{A}-{W}_{L}}$$

In this case, the sample weights in air and immersing liquid are represented by W_A_ and W_L_, respectively. The mechanical features were computed by applying the Makishima and Mackenzie concept^30,31^. The primary elements of this model, the packing density (V_t_) and dissociation energy (G_t_), are displayed below:

4 $$V_{t} = \frac{{\rho \:}}{M}\sum\nolimits_{i} {x_{i} V_{i} }$$

5 $$G_{t} = \sum\nolimits_{i} {x_{i} G_{i} }$$

The packing factor, oxides’ molar ratio, molar volume, and dissociation energy are denoted by the V_i_, x_i_, V_m_, and G_i_, respectively^32,33^.

### Monte Carlo simulation

Gamma-ray shielding properties for Dy glasses were evaluated utilizing the Monte Carlo simulation method. By applying the Monte Carlo N-Particle transport simulation code (MCNP-5)^34^ the average track length (ATL) of γ-photons within the selected cell for Dy glasses was estimated. These ATLs can be estimated by applying the F4 tally in the input file. Additionally, the MCNP-5 code was connected to the nuclear database ENDF/B-VI.8, which contains all interaction cross section (ICSs) for various energetic γ-rays with the elements constituting the prepared glasses. For accurate simulation process with low relative errors, a well description for the geometry should be listed in the input file. The literature review^35,36^ includes detailed descriptions of the cells, surfaces, materials, cutoff, and tally cards that were modified within the MCNP-5 code’s input file. After 10^8^ historical emission, the number of particles (NPS) of the cutoff card was set to kill the photon emissions. After the simulation process is finished, a new output file is immediately created with comprehensive details on the ATLs at every cell that was created in the input file, along with its relative error, which doesn’t exceed ± 0.2% in the current work. Using the simulated ATLs within the Dy glasses, the primary shielding factor (i.e., linear attenuation coefficient (LAC, cm^−1^)) was examined according to Eq. 6.

6 $$\:LAC\:\left(c{m}^{-1}\right)\:\:=\:\frac{ln\left(\frac{{I}_{o}}{{I}_{t}}\right)}{t\:\left(cm\right)}\:\:\:\:\:\:\:\:\:\:\:\:\:\:\:\:\:\:\:\:\:\:\:\:\:\:\:\:\:\:\:\:\:\:\:\:\:\:\:\:\:\:\:\:\:\:\:\:\:\:\:\:\:\:\:\:\:\:\:\:\:\:\:\:$$

The I_o_ and I_t_ in Eq. 6 refer to the average photon flux before and after interacti on with the Dy glasses. The t (cm) represents the thickness of the Dy glass sample. Additionally, the half-value thickness (HVL, cm), which specifies the thickness of the Dy glasses that can lower the It photons to 0.5 I_o_, is described. The simulated LACs for the Dy glasses can be used to calculate the HVL, according to Eq. 7^37^.

7 $$\:HVL\:\left(cm\right)=\:\frac{0.693}{LAC\:\left(c{m}^{-1}\right)}\:\:\:\:\:\:\:\:\:\:\:\:\:\:\:\:\:\:\:\:\:\:\:\:\:\:\:\:\:\:\:\:\:\:\:\:\:\:\:\:\:\:$$

The layer of the Dy samples with comparable shielding qualities to 1 cm of pure lead (Pb) is represented by the thickness equivalent lead (TEL, cm). This parameter can be derived from Eq. 8, while Eqs. 9 and 10 are used to calculate the TF and RPE:^38^.

8 $$\:TEL\:\left(cm\right)=\frac{{X}_{lead}\:(\text{ln}\left(\frac{{I}_{o}}{{I}_{t}}\right){)}_{Dy\:glass}}{(\text{ln}\left(\frac{{I}_{o}}{{I}_{t}}\right){)}_{lead}}\:\:\:\:\:\:\:\:\:\:\:\:\:\:\:\:\:\:\:\:\:\:\:\:\:\:\:\:\:\:\:\:\:\:\:\:\:\:\:\:\:\:\:\:\:\:\:\:\:\:\:\:\:\:$$

9 $$\:TF\:\left(\%\right)=\:\left(\frac{{I}_{t}}{{I}_{o}}\right)\times\:\:100\:\:\:\:\:\:\:\:\:\:\:\:\:\:\:\:\:\:\:\:\:\:\:\:\:\:\:\:\:\:\:\:\:\:\:\:\:\:\:\:\:\:\:\:\:\:\:\:\:\:\:\:\:\:\:\:\:\:\:\:\:\:\:\:\:\:\:\:\:\:\:\:$$

10 $$\:RPE\:\left(\%\right)\:=\frac{{I}_{a}}{{I}_{o}}\times\:100=(1-(\frac{{I}_{t}}{{I}_{o}}\left)\right)\:\times\:100\:\:\:\:\:\:\:\:\:\:\:\:\:\:\:\:\:\:\:\:\:\:\:\:\:\:\:\:\:\:\:\:\:\:\:$$

Additionally, the effective atomic number (Z_eff_) is a parameter that can describe the multi-element composite in term of the equivalent element. It can be calculated based on the chemical abundance (w_i_) and the atomic number (Z_i_) of i^th^ element in the fabricated glass or composite (Eq. 11).

11 $$\:{Z}_{eff}=\:\sqrt[2.94]{{w}_{1}{Z}_{1}^{2.94}+\:\:{w}_{2}{Z}_{2}^{2.94}+\dots\:+{w}_{i}{Z}_{i}^{2.94}\:\:\:\:}\:\:\:\:\:\:\:\:\:\:\:\:\:\:\:\:\:\:\:\:\:\:\:\:\:$$

## Results and discussion

### X-ray diffraction

**Fig. 2:**
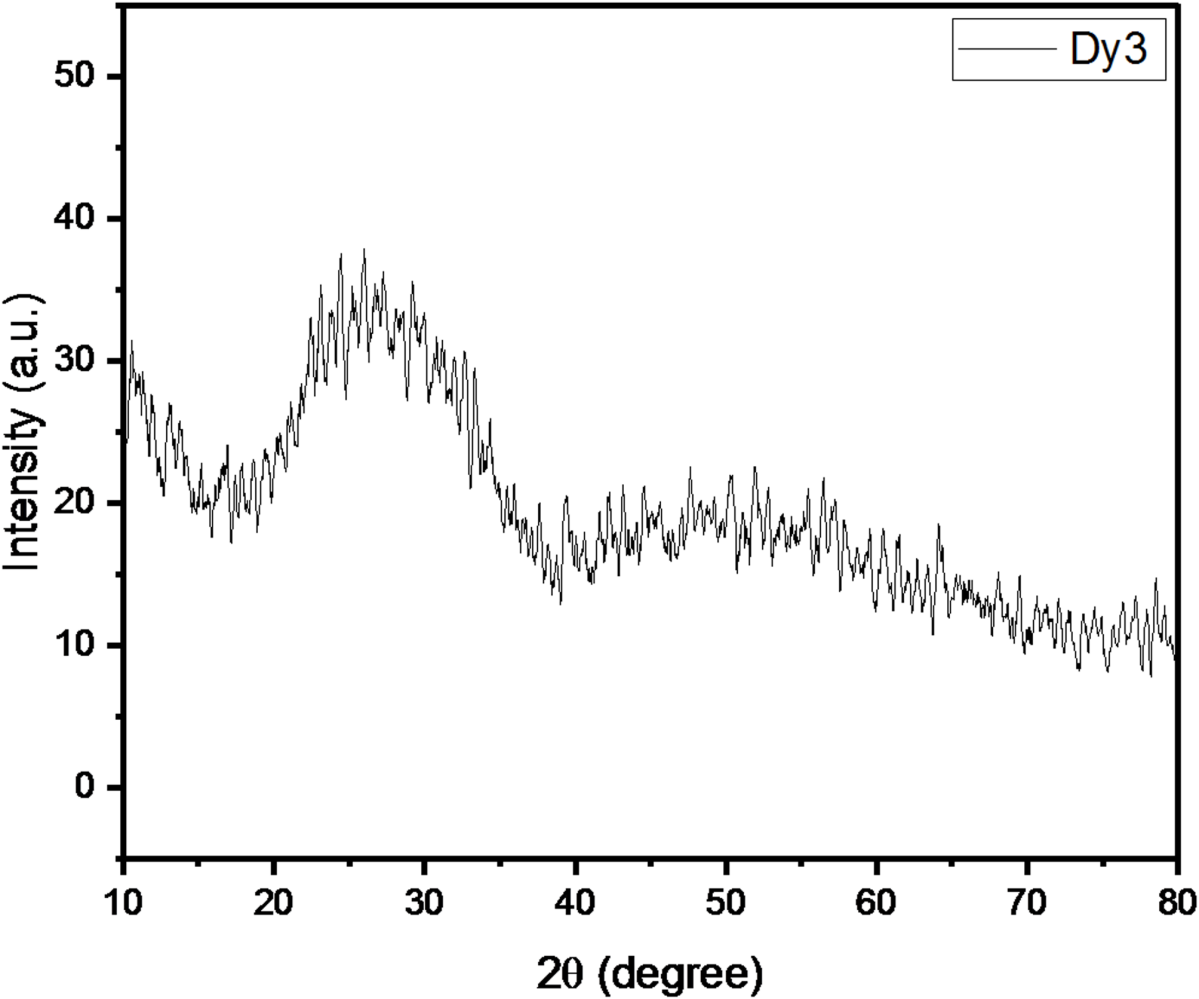
XRD results for Dy3 glasses.

The Dy3 sample’s XRD results are displayed in Fig. 2, revealing two peaks of a broad nature that prove the amorphous character of the manufactured samples by showing that the glass samples lack long order.

### Mechanical and structural properties

Any material’s mechanical qualities are essential to assess its solidity. Numerous concepts, including Makishima and Mackenzie’s^30,31^ were presented to evaluate the mechanical features of glasses theoretically. The produced glasses’ computed mechanical parameters are displayed in Table 2. One reliable measure of glass compactness is the relationship between molar volume and density. The substitution of B_2_O_3_ by Dy_2_O_3_ led to an increase in their molar volume and density for the glass system. The addition of Dy_2_O_3_ to the glasses, which resulted in an open glass structure and decreased the compactness of the sample, can be used to explain this increase. The earlier argument was further supported by the decreased predicted packing density, which showed a decrease in glass compactness. Additional measurements that provide light on glass compactness include OMV and OPD. Through the addition of a sizeable ionic radius (Dy^3+^) rather than a small ionic radius (B^3+^), which took up greater space and resulted in a structure of open glass, the OMV values for the Dy1 and Dy4 samples increased from 11.317 to 12.185 cm^3^/mol. Reducing the OPD values from 88.361 to 82.063 mol/cm^3^ for the Dy1 and Dy4 samples supports this finding.

**Table 2:** Mechanical and structural features of glass samples doped with different ratios of Dy_2_O_3_.

Mechanical and structural properties	Glass samples			
	Dy1	Dy2	Dy3	Dy4
Hardness (GPa)	5.212	5.222	5.232	5.262
Oxygen Packing Density (OPD)	88.361	85.006	83.990	82.063
Oxygen Molar Volume (OMV, cm^3^/mol)	11.317	11.763	11.906	12.185
Molar volume (V_m_, cm^3^/mol)	22.634	23.233	23.812	24.371
Poisson ratio	0.280	0.275	0.271	0.261
Packing factor (V_i_, cm^3^/mol)	14.347	14.394	14.442	14.182
Packing density (V_t_)	0.633	0.619	0.606	0.581
Young’s modulus (GPa)	91.407	89.170	87.120	83.426
Dissociation energy (kcal/cm^3^)	17.249	17.215	17.182	17.148
Bulk modulus (GPa)	69.307	66.084	63.203	58.071
Shear modulus (GPa)	38.042	37.268	36.560	35.297
Longitudinal modulus (GPa)	120.030	115.776	111.950	105.134
Fractal bond conductivity (d)	2.195	2.255	2.313	2.431

The relationship between fractal bond conductivity (d) and the Poisson ratio is displayed in Table 2. High cross-linking density glass is reported to have a Poisson ratio of less than 0.3. For the Dy1 and Dy4 glasses, the Poisson ratio for present samples varied from 0.280 to 0.261. The fractal bond conductivity (d) results for the Dy1 and Dy4 samples are in the 2.195 to 2.431 range. The above data shows that the glasses feature a network structure between two- and three-dimensional disordered networks. There was an increase in the hardness (H) results for the samples that were currently used. For instance, the Dy1, Dy2, Dy3, and Dy4 samples have H values of 5.212, 5.222, 5.232, and 5.262 GPa. This increase indicates the glass surface’s ability to withstand scratches from sharp tools.

**Fig. 3:**
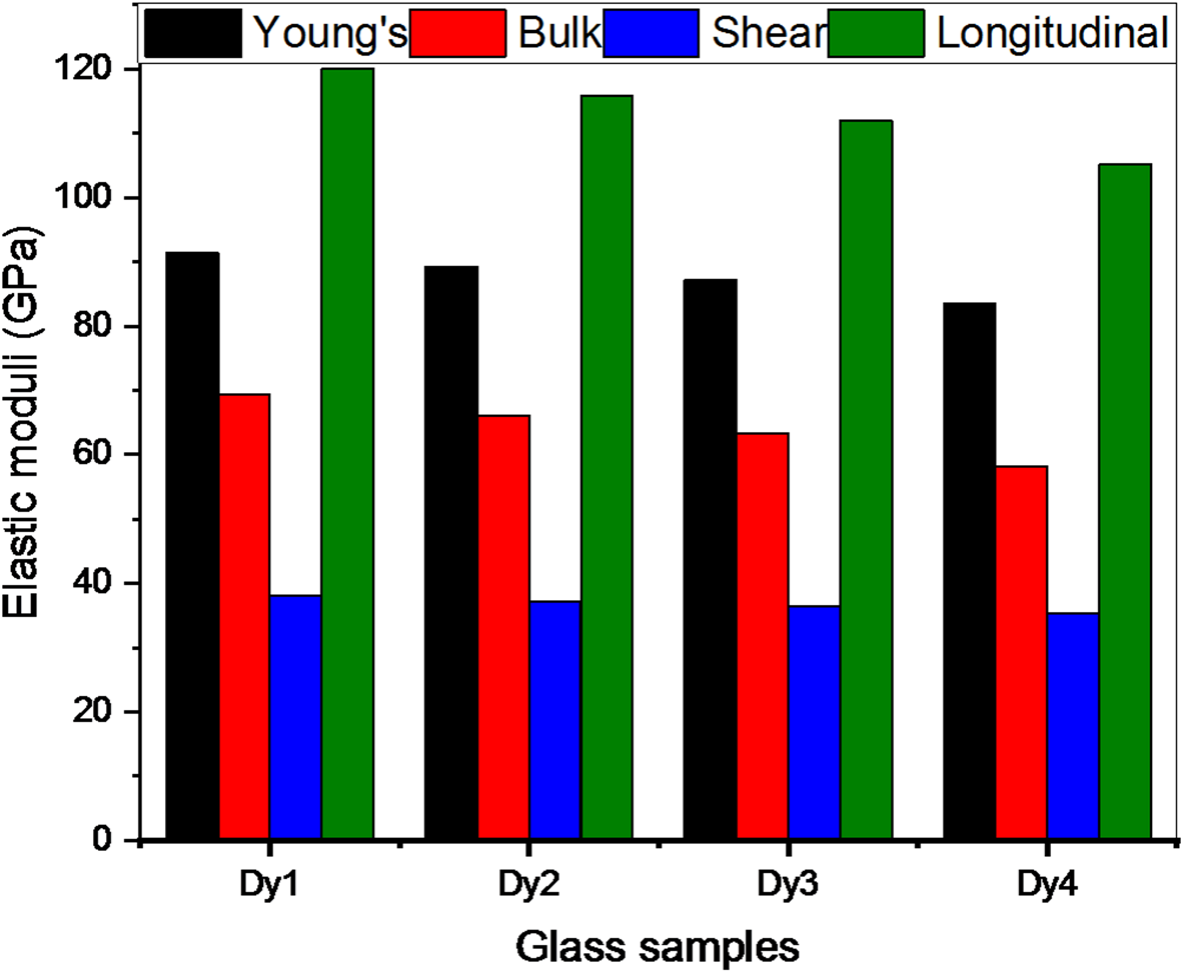
Elastic moduli of glasses doped with various ratios of Dy_2_O_3_.

**Fig. 4:**
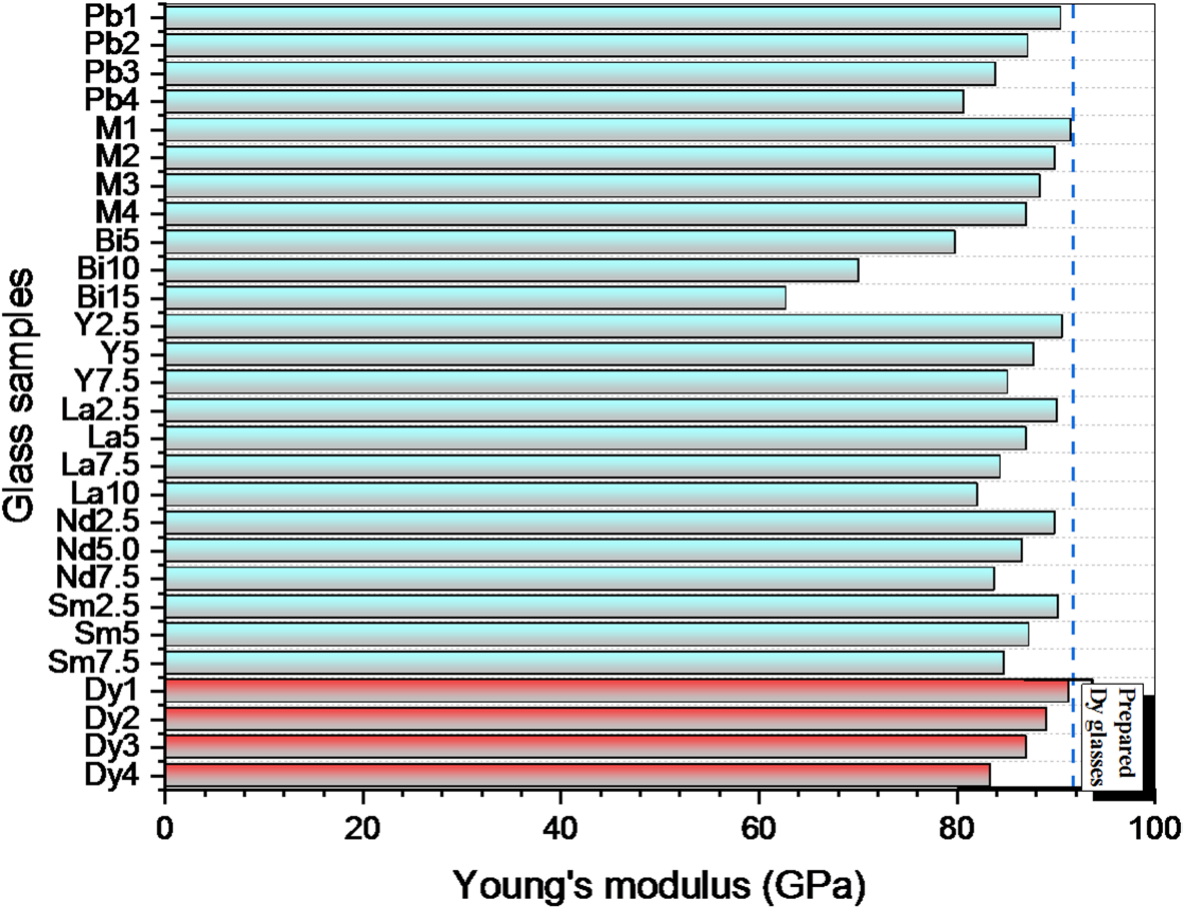
Comparison of Young’s modulus of fabricated samples with the same glass host samples doped with different oxides.

Figure 3 shows the elastic modulus for fabricated glasses. Dy_2_O_3_ was added, and all elastic moduli decreased. For instance, the Dy1, Dy2, Dy3, and Dy4 samples had Young’s modulus (Y, GPa) values of 91.407, 89.170, 87.120, and 83.426 GPa, respectively. The host system’s Young’s modulus at that time was 93.397 GPa^23^. This decrease is caused by the substitution of weak bonds for strong ones, which lowers the dissociation energy (G_t_) for glasses, which are 17.249, 17.215, 17.182, and 17.148 kcal/cm^3^ for the Dy1, Dy2, Dy3, and Dy4 samples, respectively. To assess the elastic modulus for the existing glasses, we should compare the current samples in this work with other glasses with the same host, modified with various oxides^22,39–44^ and Fig. 4 illustrates this comparison. From Fig. 4, it is worth mentioning that the Dy1 sample appeared to have a Young’s modulus higher than all samples except M1.

### Optical properties

**Fig. 5:**
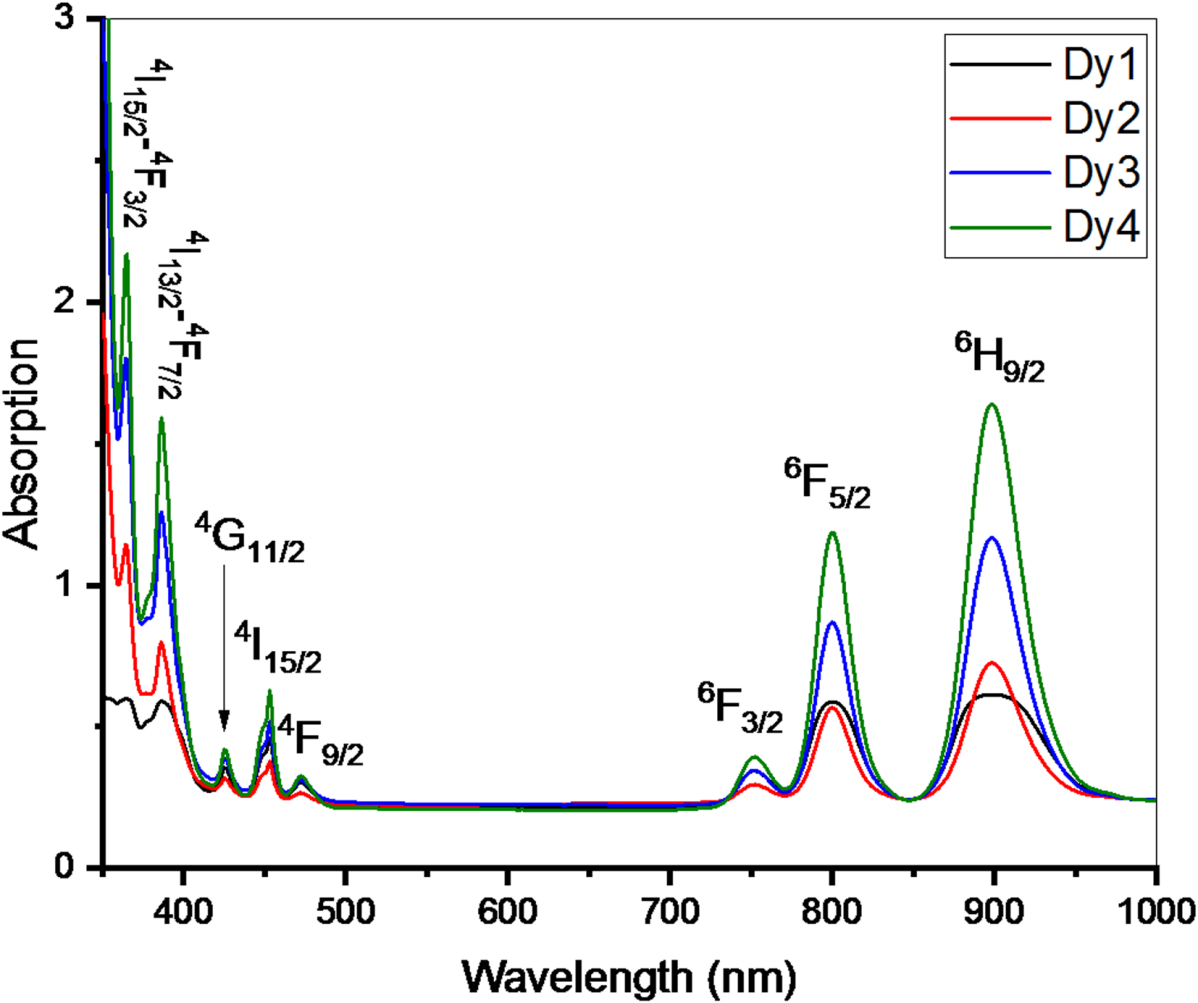
Absorption spectra of glasses doped with various ratios of Dy_2_O_3_.

The glass samples’ UV-Vis spectra in the 350 to 1000 nm wavelength range are displayed in Fig. 5. When Dy_2_O_3_ was added to the glass system, different bands formed that related to the ground state (^6^H_15/2_) to different excited state transitions: ^4^I_15/2_-^4^F_3/2_, ^4^F_13/2_-^4^F_7/2_, ^4^G_11/2_, ^4^I_15/2_, ^4^F_9/2_, ^6^F_9/2_, ^6^F_3/2_, ^6^F_5/2_, ^6^H_9/2_ which correspond to 364, 385, 424, 453, 472, 749, 800, and 898 nm, respectively^28,45^. ^4^f electron shells agree with ligands that split ion energy levels, which explains the direct association between the rare earth elements and the Coulomb field^46^. These variations in glass characteristics and the separation of energy levels dictate the absorption wavelength.

**Fig. 6:**
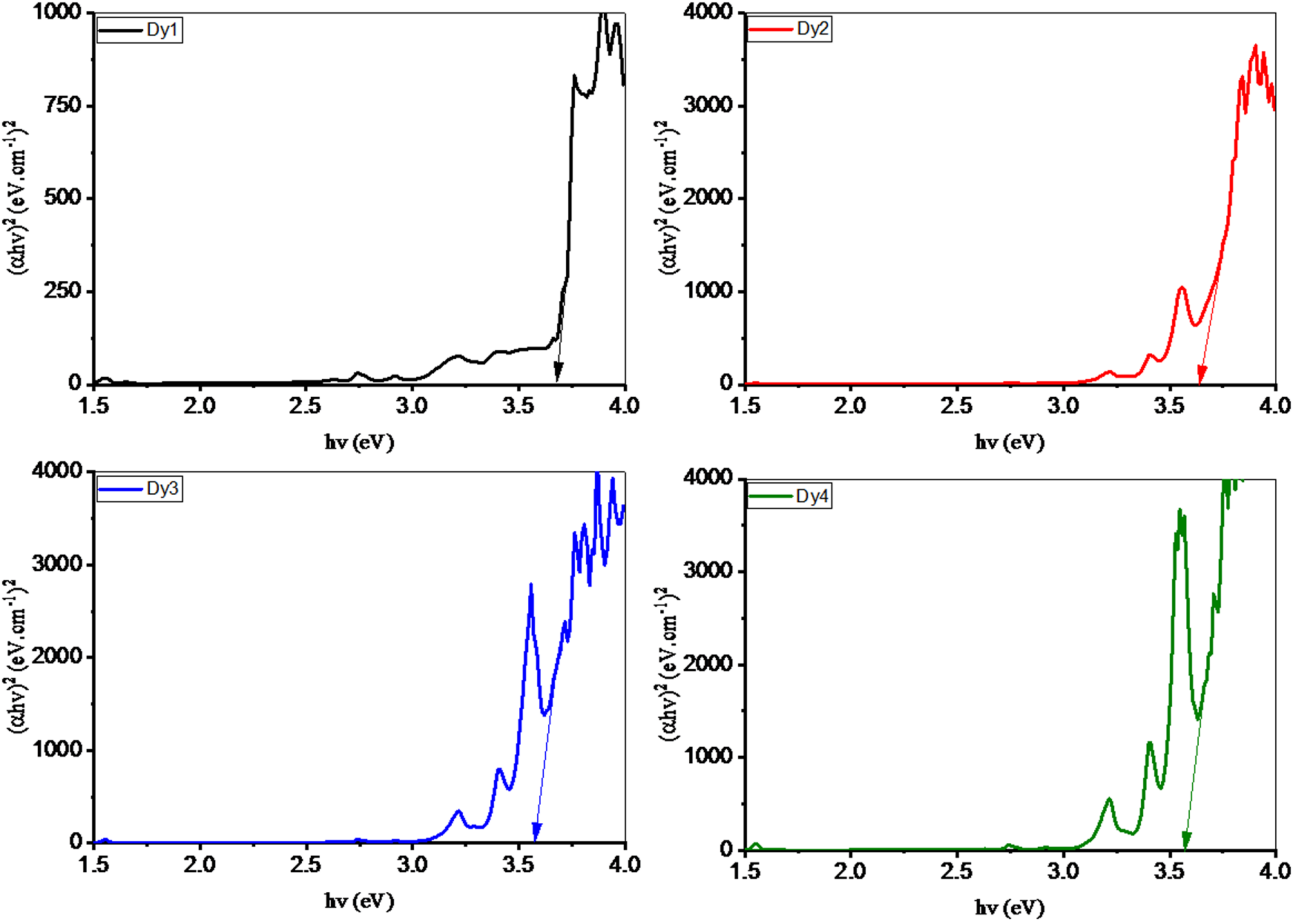
Tauc plot of glass samples doped with various ratios of Dy_2_O_3_.

The Tauc plot, determined via the Mott and Davis concept and absorption spectra, is displayed in Fig. 6 and was employed for defining the values of the band gap, as Table 3 exhibits. The measured Dy1 and Dy4 sample values dropped from 3.679 to 3.569 eV. The formation of a new valence–conduction band localization level is responsible for this decrease^47–50^. For Dy1 and Dy4 samples, the increasing polarizability and non-bridging oxygen (NBO) formation cause a 2.256 to 2.297 refractive index rise. Similarly, for Dy1 and Dy4 glasses, there was a 2.234 to 2.258 electron polarizability (α_o_) increase. However, as Table 3 shows, the rise in the refractive index resulted in the glass surface experiencing increased reflection loss. The addition of Dy_2_O_3_ progressively improved the absorption for glasses. Furthermore, for Dy1 to Dy4, the cutoff wavelength changed from low (360 nm) to high (367 nm), lowering transmission from 0.745 to 0.740. The metallization criteria (M) can be used to assess the conductive behavior of the glass sample; the semiconductor behaviors for the current samples are confirmed by the M values of 0.428, 0.426, 0.422, and 0.422 for the Dy1, Dy2, Dy3, and Dy4 samples, respectively. Finally, it is possible to observe the decrease in optical electronegativity and the increase in optical basicity. A reduction of strong glass bonding is shown by the addition of Dy_2_O_3_, which lowers optical electronegativity.

**Table 3:** Optical parameters of glass samples doped with various ratios of Dy_2_O_3_.

Optical parameters	Glass samples			
	Dy1	Dy2	Dy3	Dy4
Direct energy band gap (eV)	3.679	3.638	3.573	3.569
Refractive index (n)	2.234	2.243	2.257	2.258
Transmission	0.745	0.743	0.740	0.740
Reflection loss (R)	0.571	0.573	0.577	0.577
Metallization	0.428	0.426	0.422	0.422
Optical electronegativity (χ)	0.988	0.977	0.960	0.959
Electron polarizability (αₒ)	2.609	2.619	2.635	2.636
Optical basicity (Λ)	1.205	1.211	1.219	1.220
Cutoff wavelength (nm)	360	362	364	367

On the other hand, the rise in optical basicity suggests that ionic bonds rather than covalent ones are forming, which reduces glass stability. Here, it can be observed that the mechanical and optical features affirmed the reduction of the glass stability by adding Dy_2_O_3_.

### Gamma-ray shielding properties

**Fig. 7:**
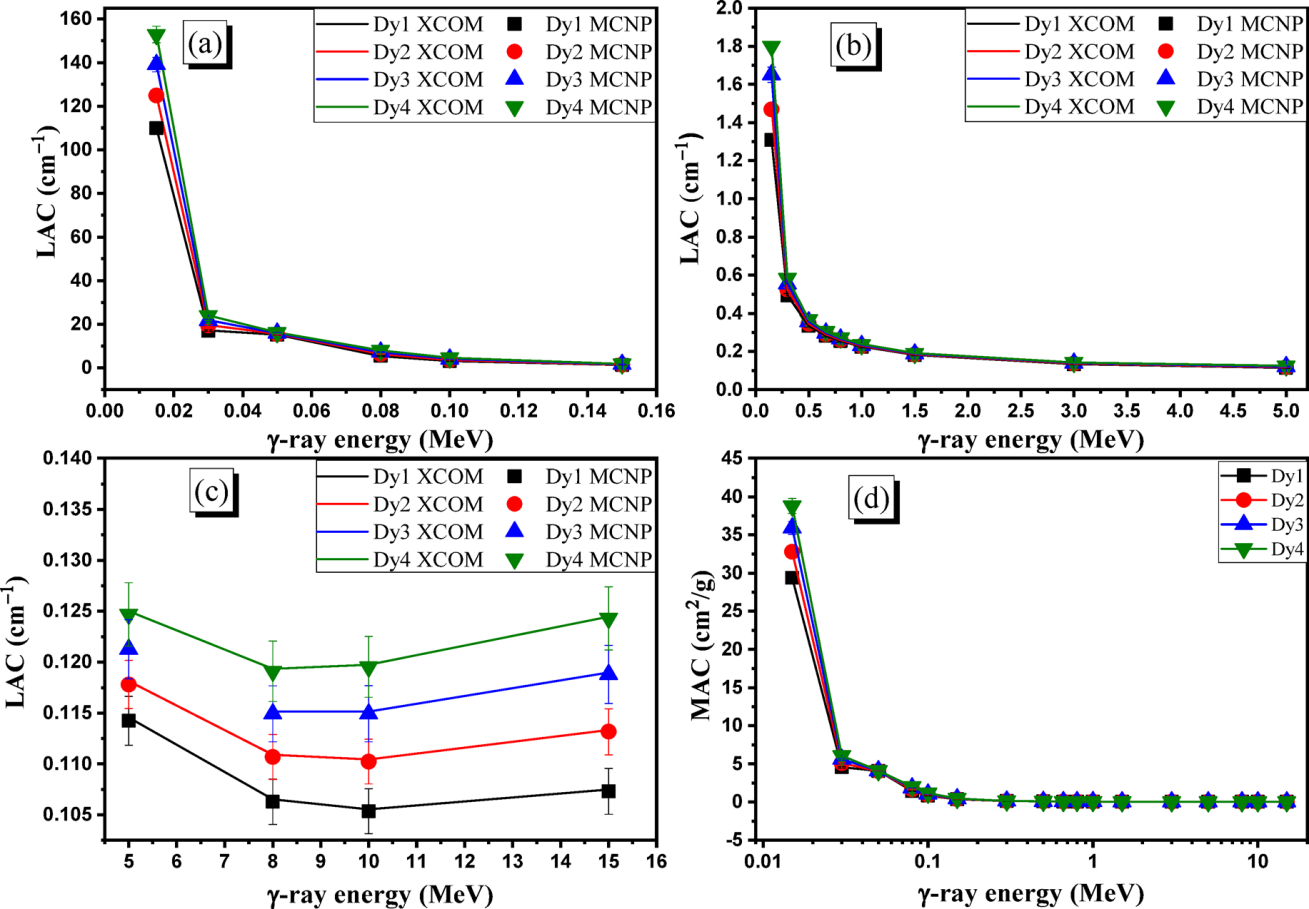
The variation of LACs (**a**) at PE interval, (**b**) CS interval, (**c**) PP interval, and (**d**) MAC (cm^2^/g) of the prepared Dy glasses as a function of the γ-ray energy (MeV).

The variation of LACs of the prepared Dy glasses as a function of the γ-ray energy was studied in Fig. 7**(a–c)**. Figure 7a shows a high reduction in the LACs of prepared Dy samples when the E_γ_ increases across the interval of 0.015–0.15 MeV. Over the aforementioned γ-ray energy interval, the LACs dropped throughout 109.882–1.309 cm^−1^ for the Dy1 sample, 124.821–1.469 cm^−1^ for the Dy2 sample, 139.096–1.650 cm^−1^ for the Dy3 sample, and 152.849–1.800 cm^−1^ for the Dy4 sample. These illustrated data show a high reduction in the LACs of prepared Dy1, Dy2, Dy3, and Dy4 by 98.81%, 98.82%, 98.81%, and 98.82%, when the E_γ_ raised across 0.015–0.15 MeV. This high reduction in the LACs is attributed to the photoelectric (PE) interaction, which is the main interaction at low energy intervals. Across the PE interaction region, the electronic interaction cross sections (ICSs) are proportional to $\:{E}_{\gamma\:}^{-3.5}$. This leads to a high reduction in the probability of interaction between γ-ray and electrons within the fabricated glass thickness. Raising E_γ_ values leads to an increase in transmitted photons (I_t_), while the I_o_/I_t_ ratio and LACs decreased. Further increases in the E_γ_ values higher than 0.15 MeV, the PE interaction becomes weak within the prepared Dy glasses compared to the CS interaction. The electronic interaction cross section within the CS region (between 0.3 and 5 MeV) fluctuates with $\:{E}_{\gamma\:}^{-1}$^51^ resulting in a moderate exponential reduction in the LACs of fabricated glasses, as seen in Fig. 7b.

The increase in E_γ_ value across the range of 0.3-5 MeV is accompanied by a reduction in the LACs throughout 0.494–0.114 cm^−1^ for the Dy1 sample, 0.524–0.118 cm^−1^ for the Dy2 sample, 0.554–0.121 cm^−1^ for the Dy3 sample, and 0.583–0.125 cm^−1^ for the Dy4 sample. The presented data showed a moderate decline in the LACs of prepared samples Dy1, Dy2, Dy3, and Dy4 by 76.9%, 77.5%, 78.1%, and 78.6%, when E_γ_ increased throughout 0.3-5 MeV.

At high γ-ray energies where E_γ_ ≥ 5 MeV, the CS interaction diminishes, while the pair production interaction (PP) becomes stronger. Due to the proportionality of ICSs along the PP interval with log (E_γ_), a slight increase in the LACs of Dy glasses is observed in Fig. 7c. The increase in E_γ_ across the range of 8–15 MeV, the LACs increase by 0.96% (from 0.106 to 0.107 cm^− 1^), 2.24% (from 0.111 to 0.113 cm^−1^), 3.37% (from 0.115 to 0.119 cm^−1^), and 4.37% (from 0.119 to 0.124 cm^−1^), for the prepared glasses Dy1, Dy2, Dy3, and Dy4, respectively. Figure 7 (a-c) shows agreement between the simulated and the calculated XCOM data over the selected Eγ interval.

After that, Fig. 7d shows the behavior of MACs for prepared Dy glasses at various E_γ_ values. The MACs presented in Fig. 7d were computed based on the LACs that were obtained using MCNP-5 code. Increasing the Eγ values over the interval of 0.015-15 MeV is accompanied by a reduction in the MACs over the intervals of 29.380–0.029 cm^2^/g (for the Dy1 sample), 32.787–0.030 cm^2^/g (for the Dy2 sample), 35.914–0.031 cm^2^/g (for the Dy3 sample), and 38.794–0.032 cm^2^/g (for the Dy4 sample). The reduction in the MACs is attributed to the same mentioned reasons illustrated in the LAC part.

In order to illustrate the potential of the prepared Dy glasses in the radiation shielding applications, their LACs were compared to the LACs of similar glasses selected from literature as well as the LACs of some commercial (Schott) radiation protective glasses, as illustrated in Table 4. The LACs of prepared Dy glasses at 0.662 MeV are 0.283 cm^−1^, 0.290 cm^−1^, 0.297 cm^−1^, and 0.304 cm^−1^ for the samples Dy1, Dy2, Dy3, and Dy4, respectively. According to the data collected in Table 4, the glass Dy1 has the lowest LAC (0.283 cm^−1^) among the fabricated Dy glasses in the current study; its LAC is close to the LACs reported for samples BaLi8 (0.278 cm^−1^)^52^ and S1 (0.279 cm^−1^)^53^. Additionally, the Dy1 glass has LACs higher than the LACs reported for BaLi1 (0.175 cm^−1^), BaLi2 (0.192 cm^−1^)^52^ BaMo1 (0.280 cm^−1^)^54^ BZLSn0 (0.226 cm^−1^), BZLSn5 (0.244 cm^−1^)^55^ BCrBi-0 (0.185 cm^−1^), BCrBi-5 (0.196 cm^−1^), BCrBi-25 (0.251 cm^−1^)^56^ ANBP00 (0.161 cm^−1^), ANBP10 (0.269 cm^−1^)^57^ SBC-B00 (0.222 cm^−1^), SBC-B35 (0.267 cm^−1^)^58^ 0 (0.165 cm^−1^), 20 (0.192 cm^−1^)^59^ X = 5 (0.202 cm^−1^)^60^ BAlNaFe0 (0.162 cm^−1^), BAlNaFe3 (0.166 cm^−1^)^61^ LiNb0 (0.180 cm^−1^)^62^ and MBTS0 (0.235 cm^−1^)^63^. Additionally, the fabricated glasses Dy2–Dy4 have LACs that match with the LACs that were reported in literature for samples BaLi9 (0.293 cm^−1^), BaMo2 (0.283 cm^−1^), BaMo8 (0.302 cm^−1^)^54^ S5 (0.300 cm^−1^)^53^ X = 15 (0.282 cm^−1^)^60^ CuO (0.305 cm^−1^), TiO_2_ (0.299 cm^−1^), CaO (0.302 cm^−1^)^64^ and LiNb12 (0.308 cm^−1^)^62^. Table 4 shows also that all fabricated glasses Dy1-Dy4 have LACs lower than the LACs reported for glasses ANBP20 (0.349 cm^−1^), ANBP50 (0.501 cm^−1^)^57^ X = 20 (0.318 cm^−1^), X = 55 (0.478 cm^−1^)^60^ Fe_2_O_3_ (0.312 cm^−1^)^64^ MBTS35 (0.347 cm^−1^), MBTS70 (0.423 cm^−1^)^63^ A1 (0.367 cm^−1^), A4 (0.433 cm^−1^)^65^ LBWB0 (0.387 cm^−1^), and LBWB5 (0.359 cm^−1^)^66^.

**Table 4:** Comparing the lacs of prepared dy glasses those of similar Borate glasses selected from literature and commercial (Schott) radiation protective glasses.

Sample	LAC (cm^−1^)	Ref	Sample	LAC (cm^−1^)	Ref
Dy1	0.283	Current glass samples	X = 5	0.202	^55^
Dy2	0.290		X = 15	0.282	
Dy3	0.297		X = 20	0.318	
Dy4	0.304		X = 55	0.478	
BaLi1	0.175	^47^	BAlNaFe0	0.162	^56^
BaLi2	0.192		BAlNaFe3	0.166	
BaLi8	0.278		Fe_2_O_3_	0.312	^59^
BaLi9	0.293		CuO	0.305	
BaMo1	0.280	^49^	TiO2	0.299	
BaMo2	0.283		CaO	0.302	
BaMo8	0.302		LiNb0	0.180	^57^
BZLSn0	0.226	^50^	LiNb12	0.308	
BZLSn5	0.244		MBTS0	0.235	^58^
BCrBi-0	0.185	^51^	MBTS35	0.347	
BCrBi-5	0.196		MBTS70	0.423	
BCrBi-25	0.251		A1	0.367	^60^
S1	0.279	^48^	A4	0.433	
S5	0.300		LBWB0	0.387	^61^
ANBP00	0.161	^52^	LBWB5	0.359	
ANBP10	0.269		RS253	0.19	^62^
ANBP20	0.349		RS253 G18	0.19	
ANBP50	0.501		RS 323 G19	0.28	
SBC-B00	0.222	^53^	RS 360	0.32	
SBC-B35	0.267		RS 520	0.5	
0	0.165	^54^			
20	0.192				

Regarding the Schott AG radiation protective glasses, Table 4 shows that the prepared sample Dy1 has LACs close to the protective glass RS 323 G19 (0.28 cm^−1^)^62^ which has around 33 wt% of PbO within its chemical composition. The fabricated glass Dy1 also has a LAC higher than the LAC reported for glasses RS253 (0.19 cm^−1^) and RS253 G18 (0.19 cm^−1^). In contrast, the fabricated glasses Dy1-Dy4 have LACs lower than the reported protective glasses RS 360 (0.32 cm^−1^) and RS 520 (0.5 cm^−1^). The high concentration of dense PbO in RS360 and RS520, which reaches 45 and 71 wt% of their composition^67^ respectively, is responsible for their high LACs.

**Fig. 8:**
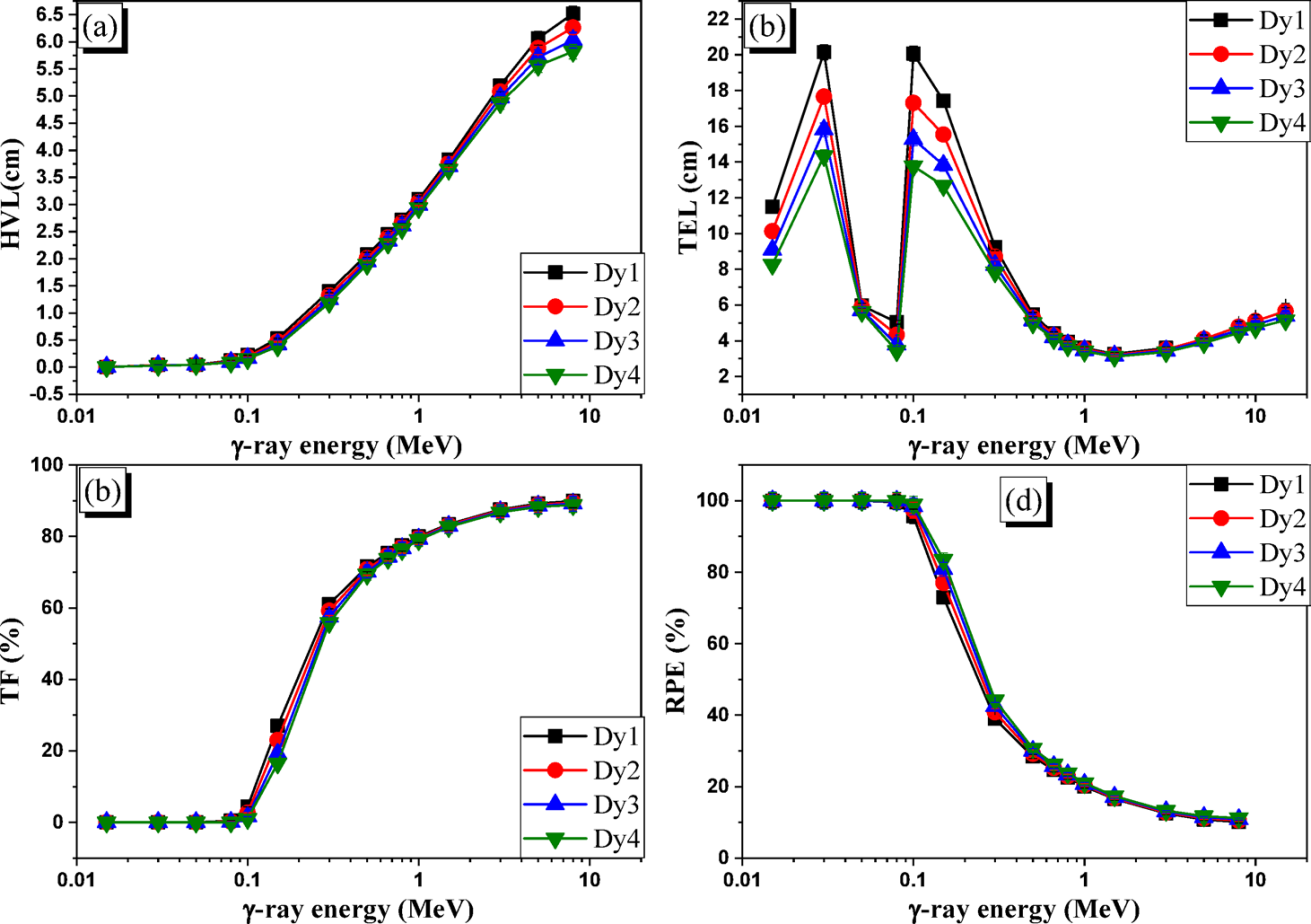
The influence of γ-ray energy on the (**a**) HVL (cm), (**b**) TEL (cm), (**c**) TF (%), and (**d**) RPE (%) of the prepared Dy glasses.

The HVL variation as a function of the E_γ_ values was shown in Fig. 8a. The increase in E_γ_ from 0.015 to 5 MeV causes HVLs to rise, while the increase in E_γ_ over an interval between 8 and 15 MeV causes HVLs of prepared glasses to fall. These aforementioned fluctuation tendencies appear because the HVLs for the Dy glasses are inversely proportional to their LACs. The data presented in Fig. 8a show that the increase in E_γ_ over 0.015-5 MeV causes the HVLs to increase over the ranges of 0.006–6.068 cm for the Dy1 sample, 0.006–5.884 cm for the Dy2 sample, 0.005–5.716 cm for the Dy3 sample, and 0.005–5.559 cm for the Dy4 sample in that order.

The increase in HVLs is due to the PE and CS interactions, representing the main interactions during the selected interval. On the other hand, as the E_γ_ values rise throughout the 8–15 MeV range, the computed HVLs fall over the following intervals: 6.522–6.460 cm for the Dy1 sample, 6.263–6.125 cm for the Dy2 sample, 6.031–5.835 cm for the Dy3 sample, and 5.820–5.576 cm for the Dy4 sample. The observed decline in the HVL across the high E_γ_ range is caused by the PP interaction. TEL variation for Dy glasses ranges from 11.497 to 5.031 cm for the Dy1 sample, 10.121 to 4.329 cm for the Dy2 sample, 9.082 to 3.780 cm for the Dy3 sample, and 8.265 to 3.426 cm for the Dy4 sample for the PE region between 0.015 and 0.8 MeV. The decrease in LACs of pure Pb, that is slightly higher than the decrease in LACs of the Dy sample with increasing the E_γ_ within the PE range, is responsible for this increase in TELs. While the LACs of the Dy1, Dy2, Dy3, and Dy4 samples decreased by 95.05%, 94.93%, 94.79%, and 94.77%, the LACs of pure Pb decreased by 97.83% when E_γ_ increased between 0.015 and 0.8 MeV.

The TELs for samples attain high values at 0.03 MeV and 0.1 MeV because of the L1 and K-edges. At 0.03 MeV, the TELs approach 20.142, 17.662, 15.802, and 14.348 cm, while at 0.1 MeV, they approach 20.062, 17.305, 15.277, and 13.771 cm. Because the LACs of Pb and Dy glasses decrease exponentially with increasing E_γ_, the TELs fell when the E_γ_ was raised across the energy region of 0.3–1.5 MeV (CS interval). A greater decrease in the LACs of pure Pb, reaching 87.05%, is accompanied by an increase in E_γ_ over the 0.3–1.5 MeV range. This reduction is greater than that of observed for the prepared glasses Dy1 (63.29%), Dy2 (64.87), Dy3 (66.21%), and Dy4 (67.35%).

The decrease in TELs throughout the interval of 9.246–3.262 cm for the Dy1 sample, 8.704–3.209 cm for the Dy2 sample, 8.240–3.158 cm for the Dy3 sample, and 7.834–3.108 cm for the Dy4 sample as the E_γ_ increased over the interval of 0.3–1.5 MeV is primarily due to the previously demonstrated decrease in LACs of Pb and prepared Dy glasses. Figure 8b indicates that the TELs for the Dy glasses rise with increasing E_γ_ within the E_γ_ interval of 3–15 MeV. In contrast to the decrease in the LACs of glasses Dy1, Dy2, Dy3, and Dy4, which approach 19.59%, 17.04%, 14.71%, and 12.59%, respectively, the large increase in Pb LACs (33.63%) is the cause of this increase in TELs. TELs for Dy1, Dy2, Dy3, and Dy4 increased over 3.592–5.969 cm, 3.514–5.660 cm, 3.441–5.391 cm, and 3.370–5.153 cm, respectively, as a result of the increase in E_γ_ over the 3–15 MeV interval.

As seen in the LAC section, a rise in E_γ_ results in a decrease in ICSs, which in turn affects electron-photon interactions. As a result, there was a decrease in the absorbed photons (I_a_) in the glass layer and a rise in the transmitted photons (I_t_). As seen in Fig. 8**(c–d)**, an increase in (I_t_) results in a drop in the RPEs for the Dy glasses and an increase in the TFs. Figure 8c illustrates how the PE interaction interval causes the TFs for synthesized Dy1, Dy2, Dy3, and Dy4 to decrease to the lowest levels at 0.015 MeV ≤ E_γ_ < 0.15 MeV. The TFs of Dy1, Dy2, Dy3, and Dy4 approach 27.01%, 23.02%, 19.20%, and 16.53%, respectively, at the conclusion of the PE interval (i.e., 0.15 MeV).

Due to the CS interaction, the (I_t_) raised when the E_γ_ was increased between 0.3 and 5 MeV. This was followed by a rise in the TFs throughout the range of 61.04 to 89.20% for the Dy1 sample, 59.19 to 88.89% for the Dy2 sample, 57.47 to 88.58% for the Dy3 sample, and 55.84 to 88.28% for the Dy4 sample. When the E_γ_ was raised between 8 and 15 MeV, the PP interaction caused the (I_t_) to slightly drop, which in turn caused the TFs for all Dy glasses to somewhat decrease. On the other hand, Fig. 8d displays the RPEs’ highest levels throughout the PE interval. As E_γ_ increases between 0.015 and 0.15 MeV, the RPEs for Dy1, Dy2, Dy3, and Dy4 glasses with a thickness of 1 cm drop between 100.00 and 72.99%, 100.00 and 76.98%, 100.00 and 80.80%, and 100.00 and 83.47%, respectively. Furthermore, when E_γ_ increased between 0.3 and 5 MeV, the RPEs for the produced glasses Dy1, Dy2, Dy3, and Dy4 with a thickness of 1 cm decreased across the interval of 38.96–10.80%, 40.81–11.11%, 42.53–11.42%, and 44.16–11.72%, respectively. The RPEs for all Dy glasses thereafter increase negligibly in tandem with the increase in E_γ_ over 8 MeV.

**Fig. 9:**
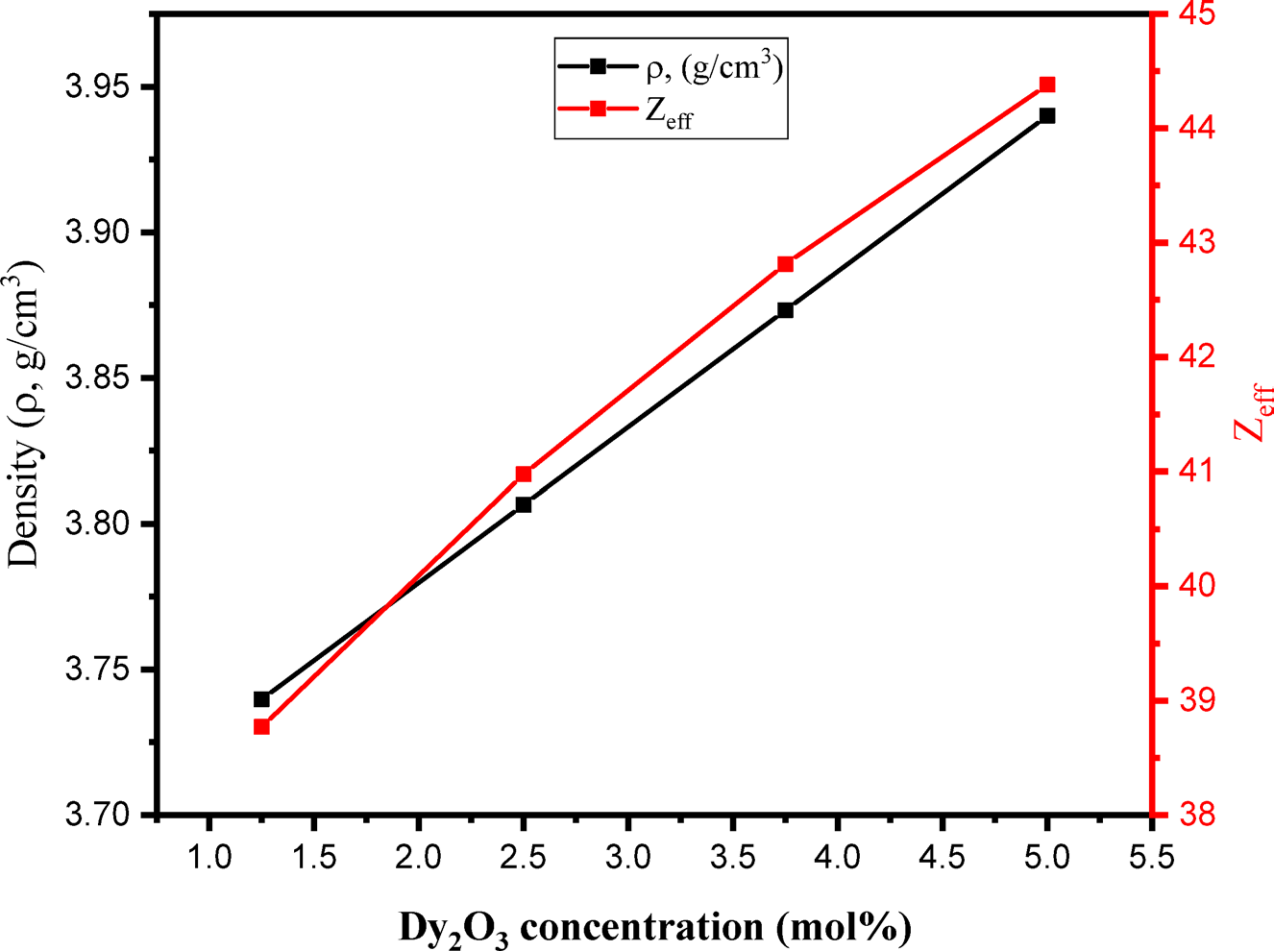
Variation of the density and Z_eff_ of the prepared samples as a function of the Dy_2_O_3_ concentration (mol%).

The chemical composition, which is represented by the replacement of B_2_O_3_ by Dy_2_O_3_, is one of the factors that affect the physical properties and γ-ray protective parameters. Regarding the physical parameters, Fig. 9 shows that the replacement of dense Dy_2_O_3_ (ρ = 7.8 g/cm^3^) for the lighter B_2_O_3_ (ρ = 2.46 g/cm^3^) increases the ρ value for the Dy glasses throughout 3.740–3.940 g/cm^3^ when the Dy_2_O_3_ concentration is raised throughout 1.25–5.0 mol%, respectively^68,69^. Furthermore, the increase in the Dy_2_O_3_-doping concentration throughout 1.25–5 mol% increases the Z_eff_ values of Dy glasses throughout 38.77–44.38, respectively, as illustrated in Fig. 9.

**Fig. 10:**
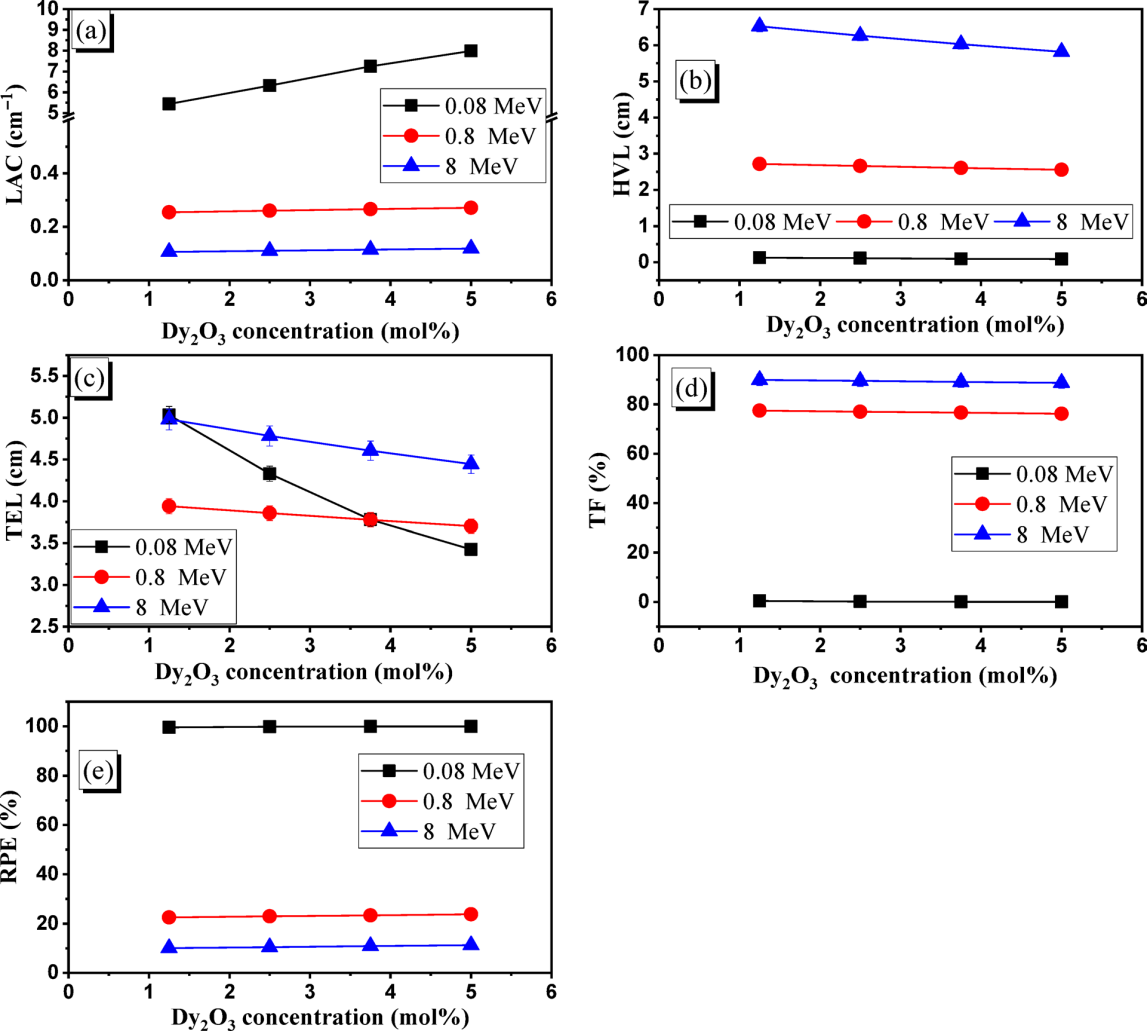
The impact of Dy_2_O_3_ concentration on the (**a**) LAC (cm^−1^), (**b**) HVL (cm), (**c**) TEL (cm), (**d**) TF (%), and (**e**) RPE (%) of the fabricated Dy glasses.

It is known that $\:{Z}_{eff}^{4.6}$, Z_eff_, and $\:{Z}_{eff}^{2}$ affect the ICSs of γ-rays within the PE, CS, and PP regions, respectively^70^. Consequently, the increase in Z_eff_ of Dy glasses is due to the increase in Dy_2_O_3_-doping concentration, which leads to a significant rise in the ICSs of γ-ray and photon-electron interactions. Consequently, as the doping concentration of Dy_2_O_3_ grew, the (I_a_) photons and RPEs rose, whereas the (I_t_) photons and TFs fell within the Dy glasses. The RPEs of the Dy glasses increase in the ranges of 10.08–11.23% at 8 MeV, 22.50–23.76% at 0.8 MeV, and 99.57–99.97% at 0.08 MeV, as shown in Fig. 10e. However, as shown in Fig. 10d, when the Dy_2_O_3_-doping concentration increased over the concentration range of 1.25–5.0 mol%, the TFs for 1 cm of Dy samples fell along ranges of 0.43–0.03% at 0.08 MeV, 77.50–76.24% at 0.8 MeV, and 89.92–88.77% at 8 MeV. Additionally, the LACs of the Dy samples were impacted by the decrease in (I_t_) photons as the Dy_2_O_3_ doping concentration increased. The LACs of Dy glasses increased between 1.25 mol% and 5 mol% as the Dy_2_O_3_ content increased, as shown in Fig. 10a. The LACs improved from 5.442 to 7.993 cm^−1^ at 0.08 MeV, 0.255 to 0.271 cm^−1^ at 0.8 MeV, and 0.106 to 0.119 cm^−1^ at 8 MeV, respectively.

Then, as shown in Fig. 10b, the HVLs of Dy glasses decrease with increasing Dy_2_O_3_-doping concentration along the range of 1.25–5.0 mol%, 2.720–2.555 cm at 0.8 MeV, and 6.522–5.020 cm at 8 MeV. The opposite relationship between HVLs and LACs for Dy glasses is the main reason for the reduction observed in the HVLs. Additionally, as Fig. 10c illustrates, the estimated TEL for Dy glasses decreased significantly when the Dy_2_O_3_-doping concentration rose due to an increase in the LACs of Dy glasses. TELs decrease along with the ranges of 5.031–3.426 cm at 0.08 MeV, 3.940–3.701 cm at 0.8 MeV, and 4.979–4.444 cm at 8 MeV when the Dy_2_O_3_ doping concentration is increased from 1.25 to 5.0 mol%. When compared to pure Pb, the addition of Dy_2_O_3_ concentration raises the LACs of Dy glasses, which lowers TELs at different E_γ_.

The study concludes that substituting Dy_2_O_3_ for B_2_O_3_ improves the radiation shielding efficiency of the produced glasses. As a result, the Dy4 sample has the best shielding parameters, whereas Dy1 has the lowest shielding performance among the synthetic glasses. The shielding performance of the prepared glasses is in the following order: Dy4 > Dy3 > Dy2 > Dy1.

## Conclusion

In conclusion, highly transparent glass groups have been made with the composition of (35-x) B_2_O_3_ + 10GeO_2_ + 20TeO_2_ + 35MgO + x Dy_2_O_3_, where x = 1.25, 2.5, 3.75, and 5 mol%. The addition of Dy_2_O_3_ to the G-T-B glass slightly changes the color of prepared glasses from nearly transparent to light yellow. The substitution of B_2_O_3_ with the light yellow Dy_2_O_3_ is the main reason for the change in the color of fabricated G-T-B glasses. These XRD peaks prove the amorphous character of the prepared glasses by showing that the glass samples lack long order. The Poisson ratio for present samples varied from 0.280 to 0.261. For the Dy1 and Dy4 samples. For instance, the Dy1, Dy2, Dy3, and Dy4 samples have H values of 5.212, 5.222, 5.232, and 5.262 GPa. The increasing polarizability and formation of non-bridging oxygen (NBO) cause a rise in refractive index from 2.256 to 2.297. Furthermore, the increase in Dy_2_O_3_ doping concentrations has a great effect on the radiation shielding parameters of the glasses. The data received from the Monte Carlo simulation reveals that the increase in Dy_2_O_3_ across the concentration of 1.25–5 mol% increases the LAC of the prepared glasses by 39.10%, 37.50%, 4.96%, and 15.84% over the intervals of 109.882–152.849 cm^−1^ (at 0.015 MeV), 1.309–1.800 cm^−1^ (at 0.15 MeV), 0.181–0.190 cm^−1^ (at 1.5 MeV), and 0.107–0.124 cm^−1^ (at 15 MeV), respectively. The prepared samples showed high shielding capacities compared to the commercial radiation shielding glasses, which are rich with PbO contents. Therefore, the prepared glasses are lead-free glasses that can be used in radiation protection applications.

## Data Availability

All data generated or analyzed during this study are included in this published, any row data will be available upon request.
